# Drivers of inequality in disability-free expectancy at birth and age 85 across space and time in Great Britain

**DOI:** 10.1136/jech-2014-204083

**Published:** 2014-06-06

**Authors:** Pia Wohland, Phil Rees, Clare Gillies, Seraphim Alvanides, Fiona E Matthews, Vikki O'Neill, Carol Jagger

**Affiliations:** 1 Institute for Ageing and Health, Newcastle University, Newcastle upon Tyne, UK; 2 School of Geography, University of Leeds, Leeds, UK; 3 Department of Health Sciences, University of Leicester, Leicester, UK; 4 Geography and Built Environment, Northumbria University, Newcastle upon Tyne, UK; 5 MRC Biostatistics Unit, Institute of Public Health, Cambridge, UK

**Keywords:** Health Status, Mortality, Ageing, Inequalities, Geography

## Abstract

**Background:**

Although mortality and health inequalities at birth have increased both geographically and in socioeconomic terms, little is known about inequalities at age 85, the fastest growing sector of the population in Great Britain (GB).

**Aim:**

To determine whether trends and drivers of inequalities in life expectancy (LE) and disability-free life expectancy (DFLE) at age 85 between 1991 and 2001 are the same as those at birth.

**Methods:**

DFLE at birth and age 85 for 1991 and 2001 by gender were calculated for each local authority in GB using the Sullivan method. Regression modelling was used to identify area characteristics (rurality, deprivation, social class composition, ethnicity, unemployment, retirement migration) that could explain inequalities in LE and DFLE.

**Results:**

Similar to values at birth, LE and DFLE at age 85 both increased between 1991 and 2001 (though DFLE increased less than LE) and gaps across local areas widened (and more for DFLE than LE). The significantly greater increases in LE and DFLE at birth for less-deprived compared with more-deprived areas were still partly present at age 85. Considering all factors, inequalities in DFLE at birth were largely driven by social class composition and unemployment rate, but these associations appear to be less influential at age 85.

**Conclusions:**

Inequalities between areas in LE and DFLE at birth and age 85 have increased over time though factors explaining inequalities at birth (mainly social class and unemployment rates) appear less important for inequalities at age 85.

## Introduction

Life expectancy (LE) in Great Britain (GB), as in many other countries, has risen rapidly over the last two decades, from 73.9 in 1981 to about 80.9 in 2011.^1^ ^2^ This has led to the belief that the GB population is ageing healthily, yet health inequalities have been evident for many years and appear not only to persist but to be increasing.^3^ The recent Marmot review has documented the causes and current state of inequality, with a focus on England,^4^ and stresses the importance of poverty, characterised by income deprivation and lack of access to a good environment, over unhealthy lifestyle choices, which are assumed to be determined by socioeconomic factors.^4–6^ Though comprehensive in its coverage of causes, the Marmot review gives little information on inequalities in very late life, despite the very old (85+) having the most rapid growth of any age group, between 1981 and 2007 doubling their share from 1.1% of the total population to 2.1%,^7^ with this trend likely to continue.^8^ In addition, late old age is the time when ill-health and disability are most common, as evidenced by results from the 2011 census where 83% of people aged 85+ in England and Wales reported a long-term health problem.^9^

With population ageing and the growth in numbers of the very old, we can no longer assume that LE increases will necessarily mean better population health overall. Health expectancies such as disability-free life expectancy (DFLE) are gaining importance for monitoring trends and inequalities in population health, as they combine information on both mortality and morbidity.^10^ The Office for National Statistics (ONS) regularly reports DFLE at birth and age 65 for the UK, its constituent countries and lower-level geographies. Continued monitoring of trends and inequalities in DFLE as well as LE are crucial to evaluate the impact of national and local policy to reduce inequalities, this being high on the government agenda,^11^ and this is particularly pertinent for GB where, even though health overall is continuously improving, inequality seems to persist.^12^ In this paper, we examine inequalities in DFLE at birth and age 85 across local areas (LAs) in GB, noting how they have changed between 1991 and 2001, and investigating potential drivers of the observed inequalities. We specifically answer the following research questions: (1) Were the changes over time in LE and DFLE at age 85 similar to those at birth? (2) Were the patterns of inequality for LE and DFLE at age 85 of a similar magnitude to those at birth in 1991 and 2001? (3) Do the same socioeconomic, sociodemographic and environmental factors explain the observed inequalities in LE and DFLE at birth in 2001 as in 1991, and do they have a similar impact on LE and DFLE at age 85?

## Methods

LE by age, gender and LAs in GB in 1991 and 2001 were calculated using standard abridged period life table methodology, with mid-year population estimates and vital statistics deaths data for 1991 and 2001.^13^ DFLE were calculated using the Sullivan method,^10^ ^14^ which requires only the age-specific and sex-specific prevalence of the health state of interest (here *disability*) from a cross-sectional study and alongside a period life table for the same time interval.

### Disability measure

Age-specific and sex-specific prevalence rates for disability were computed from the National Census limiting long-term illness (LLTI) question in 1991 and 2001. However, the questions varied somewhat between 1991 and 2001 (see online supplementary material box 1), resulting in under-reporting in 1991.^15^ To overcome this, a Brass relational model^16^ was used, the latter being based on a mathematical relationship between a ‘standard’ mortality distribution and that observed in any population. Marshall^15^ adapted the model to align 1991 to 2001 LLTI prevalence rates at a subnational level with the assumption that the national impact of the question change is applicable subnationally. Thus, gender-specific linear models were fitted relating the logit of the national prevalence of LLTI in 2001 in a particular age group to that for 1991; the resulting intercept and slope from these models then being applied to the logit of the subnational prevalence of LLTI in 1991 to get the aligned prevalence of LLTI in 1991. These aligned rates were then used to calculate 1991 DFLE. We also aligned the 1991 data set to 2001 geography,^17^ to account for the boundary changes that took place between 1991 and 2001.^17^

### Socioeconomic, sociodemographic and environmental factors

To explain inequalities, we followed a previous analysis^18^ and included unemployment, social class, ethnicity, retirement migration and population density (details in online supplementary material table S1). We added two further factors measuring rurality and deprivation. For urban–rural gradient (rurality), we adapted the sixfold DEFRA classification for England^19^ using the same non-overlapping population density groups and classifying Scottish and Welsh LAs in the same way (see online supplementary material table S1). We used the Townsend index of deprivation^20^ to group LAs into deprivation quintiles (see online supplementary material table S1) and when comparing LAs over time fixed their deprivation quintile membership to 2001 groupings.

### Statistical analysis

To describe inequalities, we present summary statistics (mean, 10th and 90th centiles, difference between 10th and 90th centiles) of male and female LE and DFLE at birth and age 85 across LAs. We then ranked LAs on LE and DFLE by year, gender and age (birth and age 85), classified them into area quintiles and visually present these quintiles in a population cartogram^21^ (see online supplementary material figures S1 and S2). To assess changes over time in inequalities in DFLE by deprivation (rurality), we fitted models with time and deprivation (rurality) and their interactions; if interaction was significant, we inspected time differences within deprivation (rurality) groups.

To investigate the relationship between LE and DFLE at birth and age 85, in both 1991 and 2001 as well as area-specific socioeconomic, sociodemographic and environmental factors, we used linear regression models (for LE) and meta-regression models (for DFLE) to allow inclusion of uncertainty around the DFLE estimates,^22^ fitting factors first singly and then in a multivariable model, and fitting separate models for men and women. We used permutation tests to adjust the significance for the inflated false-positive rates to which meta-regression analyses are susceptible.^23^ We did not undertake formal tests for outliers or influential data points, but visually evaluated scatter plots to ascertain whether trends were the results of just a few data points. The meta-regression analysis is constrained to England and Wales since retirement migration data for Scotland were not available in 2001 in the same format.

## Results

### Change in LE and DFLE at birth and age 85 between 1991 and 2001

Much has already been published on changes over time in LE and DFLE at birth, but we repeat results briefly for comparison with age 85. Between 1991 and 2001, mean LE at birth increased (women 1.7 years; men 2.5 years) with the gender gap decreasing by 0.8 years but increases in DFLE were much smaller (women 0.6 years; men 1.1 years), with the gender gap again decreasing, by 0.5 years (table 1). Over the same period inequalities in LE, as measured by the difference between the 10th and 90th centile in the ordered LA distribution, increased (women 0.3 years; men 0.4 years) and inequalities in DFLE increased even more (women 1.5 years; men 1.3 years).

**Table 1:** Life expectancies (LE) and disability-free life expectancies (DFLE) for women and men at birth and age 85 in 1991 and 2001: mean, CI and ranges expressed as percentiles of local authority areas

	1991				2001			
	LE_0_	LE_85_	DFLE_0_	DFLE_85_	LE_0_	LE_85_	DFLE_0_	DFLE_85_
Women								
Mean	79.1	6.2	63.8	1.4	80.8	6.4	64.4	1.4
CI	(79.0 to 79.2)	(6.2 to 6.3)	(63.5 to 64.0)	(1.4 to 1.5)	(80.7 to 80.9)	(6.4 to 6.5)	(64.1 to 64.7)	(1.4 to 1.5)
% change compared with 1991*					2.2	3.4	1.0	−0.7
10%†	77.3	5.4	60.1	1.1	78.9	5.7	59.8	1.0
90%†	80.7	7.0	66.9	1.8	82.6	7.2	68.2	1.8
10–90% range	3.4	1.6	6.8	0.7	3.7	1.5	8.3	0.8
90/10%	1.0	1.3	1.1	1.6	1.0	1.3	1.1	1.8
Men								
Mean	73.7	4.9	60.8	1.6	76.2	5.4	61.9	1.6
CI	(73.5 to 73.9)	(4.9 to 5.0)	(60.5 to 61.1)	(1.6 to 1.6)	(76.0 to 76.4)	(5.3 to 5.4)	(61.6 to 62.3)	(1.6 to 1.6)
% change compared with 1991*					3.4	9.3	1.9	1.3
10%†	71.5	4.1	56.4	1.2	73.9	4.7	56.8	1.2
90%†	75.6	5.7	64.5	2.0	78.4	6.1	66.2	2.0
10–90% range	4.1	1.6	8.1	0.7	4.5	1.4	9.4	0.8
90/10%	1.1	1.4	1.1	1.6	1.1	1.3	1.2	1.6

*% change not always apparent from data shown due to rounding.

†Percentile values for distribution of LAs.

LAs, local areas.

Similar to LE at birth, LE at age 85 also increased between 1991 and 2001 (women 0.2 years; men 0.5 years) but the percentage changes (women 3.4%; men 9.3%) were greater than those at birth (women 2.2%; men 3.4%) (table 1). In contrast, DFLE at age 85 hardly changed for women or men (table 1). Unlike LE and DFLE at birth, inequalities in LE at age 85 in 1991 and 2001 exceeded those in DFLE at age 85 similarly for women and men, with a slight fall between 1991 and 2001 in LE and a small increase in DFLE.

### Spatial patterning of LE and DFLE at birth and age 85

**Figure 1:**
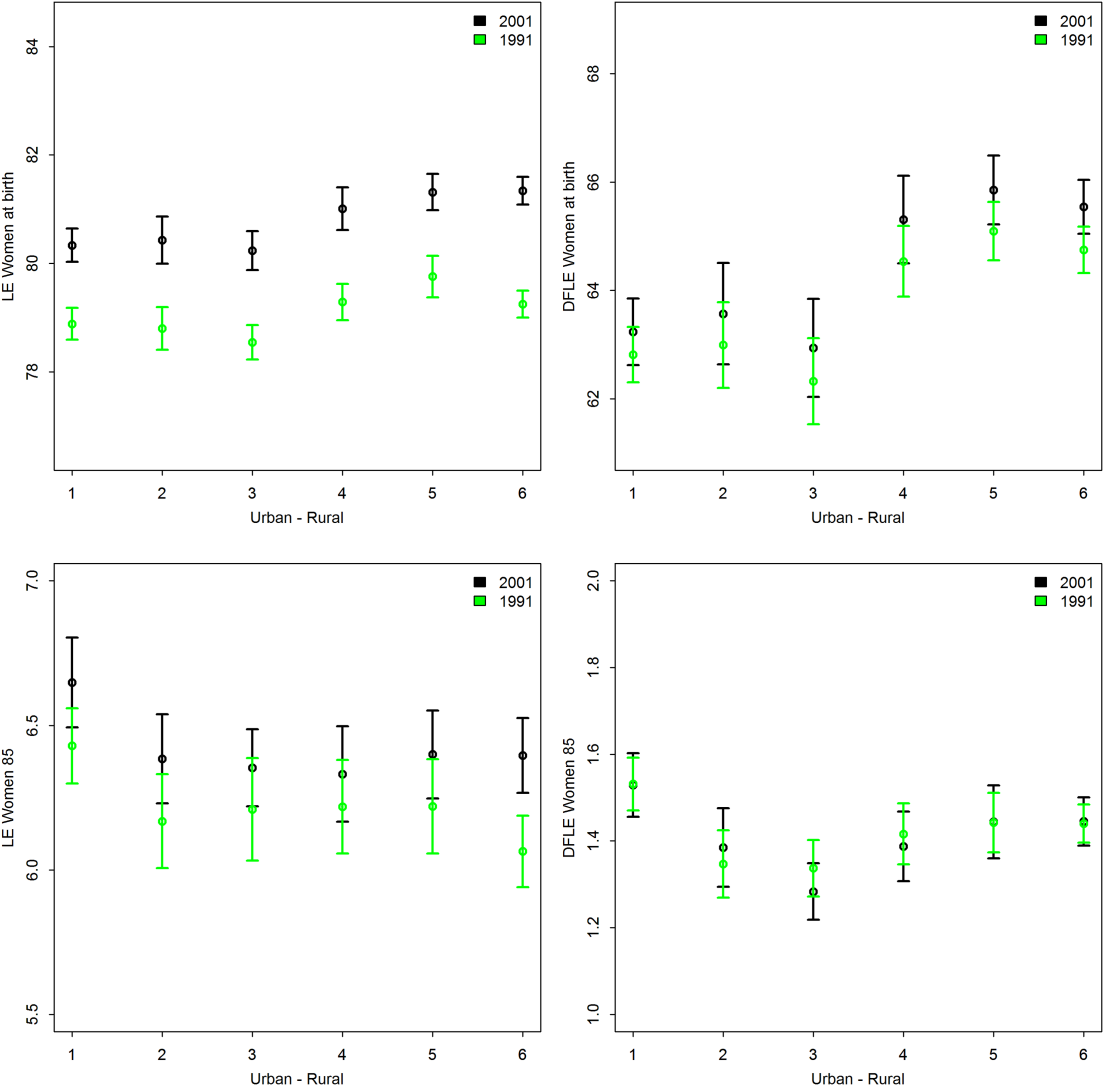
Mean life expectancy (LE) and mean disability-free life expectancy (DFLE), women, at birth and age 85, 1991 and 2001, by urban—rural classification.1=most urban, 6=most rural. Note: Y-axes do not start at 0. Y-axes starting point vary between graphs, but the length of the y-axes at birth is same for LE and DFLE and the length of the y-axes at age 85 are the same for LE and DFLE.

In 1991 and 2001, LE and DFLE for women and men at birth showed a distinct northwest to southeast gradient, commonly known as the North-South divide,^24^ though this pattern was less apparent for LE and DFLE at age 85 (see online supplementary material figures S1 and S2). For both men and women, there was no significant change over time across the urban–rural gradient (the interaction between the urban–rural gradient and time) for any of the health outcomes, LE and DFLE at birth and age 85 (see online supplementary material table S2). Nevertheless, the main effect of urban–rural gradient was significantly associated with health outcomes at birth, with, on average, an increase of one point on the urban–rural scale LE at birth increased by 0.18 years for women and 0.34 years for men while DFLE at birth increased by about half a year for both women and men (figure 1, see online supplementary material figure S3 and online supplementary material table S2).

**Figure 2:**
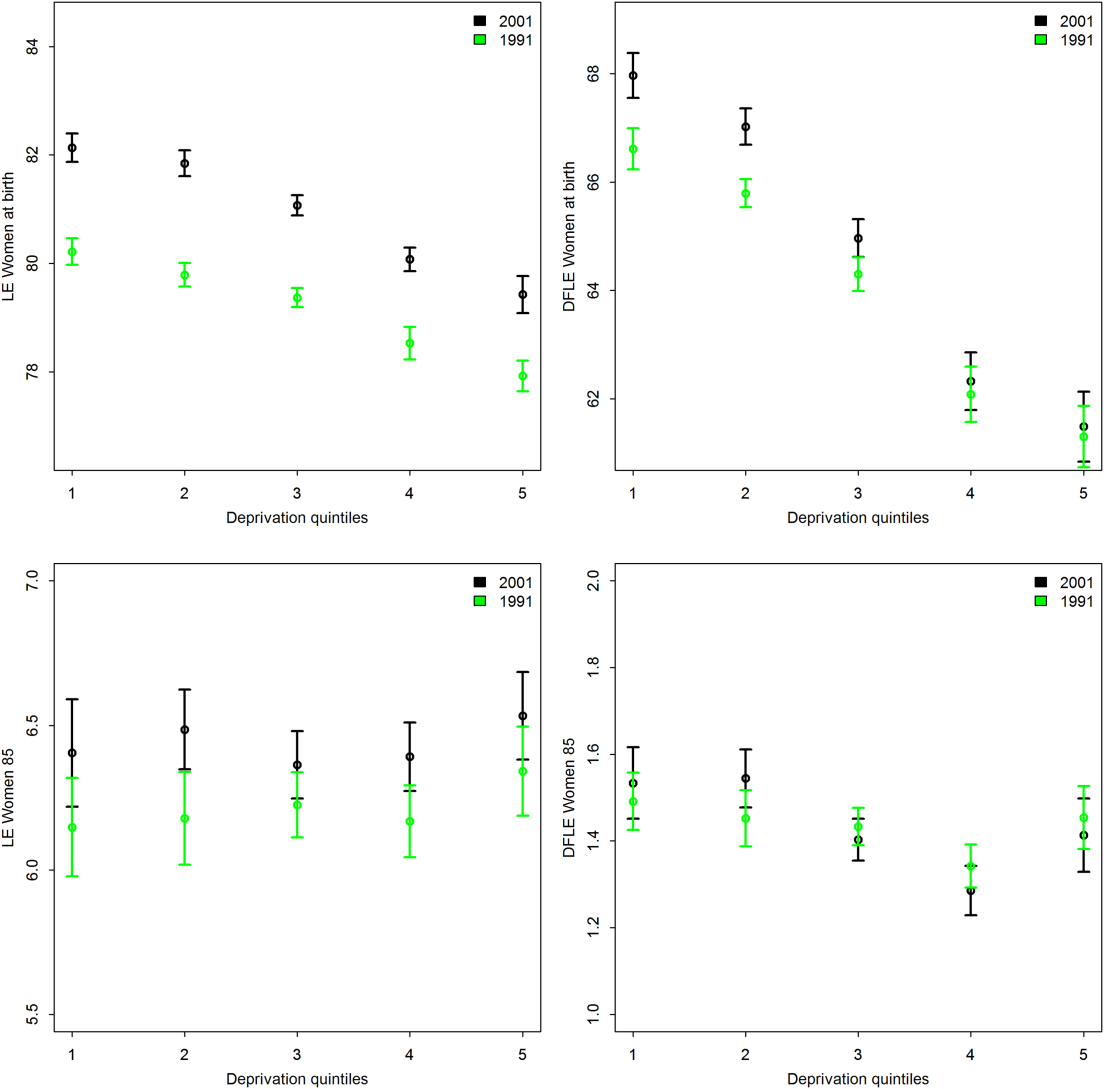
Mean life expectancy (LE) and disability-free life expectancy (DFLE), women, at birth and 85, 1991 and 2001, by deprivation quintiles applying the Townsend score 2001.1=most affluent, 5=most deprived. Note: Y-axes do not start at 0. Y-axes starting point vary between graphs, but the length of the y-axes at birth is same for LE and DFLE and the length of the y-axes at age 85 are the same for LE and DFLE.

With regards to deprivation, for both 1991 and 2001, there was a strong decline in LE and DFLE at birth from the least-deprived towards the most-deprived quintiles for women (figure 2) and men (see online supplementary material figure S4), with the gradient being steeper for DFLE compared with LE. Moreover, the significant interaction between deprivation and time for both LE at birth and DFLE at birth indicated a strengthening of the associations with deprivation over time. That is, an increase of one deprivation quintile infers a decrease in LE of 0.55 (women) and 0.82 (men) years in 1991, which decreased further in 2001 to 0.69 years (women) and 0.97 years (men). For DFLE, we found a decline of 1.34 (women) and 1.60 (men) years in 1991, which increased further to 1.65 (women) and 1.88 (men) years in 2001. When changes over time within deprivation quintiles were examined, the increase in LE was somewhat larger for more affluent areas (1.99 years for women, 2.82 years for men between 1991 and 2001) than for the most-deprived areas (1.43 for women, 2.22 years for men between 1991 and 2001) and with no significant increase in DFLE at birth in the most-deprived area quintiles (4 and 5) for men and women (figure 2 and see online supplementary material table S2).

In contrast, there was little evidence of a relationship between deprivation and LE at age 85, although the relationship with DFLE at age 85 remained as well as the significant interaction between deprivation and time, again suggesting a strengthening of the association between deprivation and DFLE over time on average for both men and women.

### Drivers of inequalities in LE and DFLE at birth and age 85, 1991 and 2001 area characteristics

We first explored the same drivers of inequalities in LE and DFLE that had been considered by Bone *et al*^18^: social class composition, unemployment rate, retirement potential, population density and ethnic composition. When considered individually, all area-level indicators explained variations in LE and DFLE at birth by LAs (table 2). The variables with the highest impact on LE and DFLE at birth were social class composition, unemployment rate and retirement potential of an area. In 1991, LE at birth decreased by about 2 months (women) and 3 months (men) and DFLE by about half a year (men and women) for each 1% increase of population social classes VI and V in an area. This gradient was somewhat steeper for unemployment rate, almost 3 months (women) and 4 months (men) in LE and 8 months (women and men) in DFLE were lost for each % increase in unemployment rate. On the other hand, retirement potential had a positive association with LE and DFLE. The standardised regression coefficients (β/SE) for both these variables increased over the decade. Actual life years and disability-free life years lost doubled between 1991 and 2001 for men and women for each % point increase in unemployment.

**Table 2:** Explanatory simple regression and meta-regression results for LE and DFLE at birth, 1991 and 2001, by gender

	LE at birth										DFLE at birth									
	1991					2001					1991					2001				
	Univariate		Multivariate			Univariate		Multivariate			Univariable		Multivariable			Univariable		Multivariable		
	β (SE)	p	β (SE)	Beta	p	β (SE)	p	β (SE)	Beta	p	β (SE)	p	β (SE)	Beta	p	β (SE)	p	β (SE)	Beta	p
Women																				
Social class IV and V (%)	−0.17 (0.01)	**<0.001**	−0.08 (0.02)	−0.25	**<0.001**	−0.24 (0.01)	**<0.001**	−0.15 (0.02)	−0.42	**<0.001**	−0.46 (0.02)	**<0.001**	−0.16 (0.03)	−0.24	**<0.001**	−0.66 (0.02)	**<0.001**	−0.35 (0.03)	−0.43	**<0.001**
Unemployment rate (%)	−0.22 (0.02)	**<0.001**	−0.18 (0.03)	−0.45	**<0.001**	−0.46 (0.03)	**<0.001**	−0.14 (0.05)	−0.19	**0.007**	−0.64 (0.03)	**<0.001**	−0.53 (0.05)	−0.65	**<0.001**	−1.33 (0.05)	**<0.001**	−0.67 (0.08)	−0.41	**<0.001**
Retirement migration	0.56 (0.06)	**<0.001**	0.06 (0.08)	0.05	0.413	1.36 (0.10)	**<0.001**	0.79 (0.09)	0.33	**<0.001**	1.71 (0.11)	**<0.001**	0.42 (0.11)	0.15	**<0.001**	3.18 (0.24)	**<0.001**	1.42 (0.15)	0.25	**<0.001**
Population density	−0.01 (0.00)	**<0.001**	0.01 (0.01)	0.12	0.101	−0.02 (0.00)	**<0.001**	0.00 (0.00)	−0.01	0.923	−0.04 (0.01)	**<0.001**	0.02 (0.01)	0.14	**0.005**	−0.04 (0.01)	**<0.001**	−0.01 (0.01)	−0.04	0.337
Non-white population (%)	−0.03 (0.01)	**0.006**	0.00 (0.01)	0.02	0.760	−0.03 (0.01)	**<0.001**	0.00 (0.01)	0.03	0.59	−0.08 (0.02)	**<0.001**	0.03 (0.02)	0.08	0.063	−0.07 (0.02)	**<0.001**	0.05 (0.01)	0.14	**<0.001**
Constant			81.8					82.9					69.6					71.3		
r^2^			0.38					0.58					0.7					0.81		
Men																				
Social class IV and V (%)	−0.22 (0.02)	**<0.001**	−0.10 (0.02)	−0.27	**<0.001**	−0.31 (0.02)	**<0.001**	−0.17 (0.02)	−0.39	**<0.001**	−0.54 (0.03)	**<0.001**	−0.19 (0.03)	−0.25	**<0.001**	−0.73 (0.03)	**<0.001**	−0.33 (0.03)	−0.37	**<0.001**
Unemployment rate (%)	−0.34 (0.02)	**<0.001**	−0.18 (0.04)	−0.35	**<0.001**	−0.66 (0.03)	**<0.001**	−0.26 (0.05)	−0.3	**<0.001**	−0.75 (0.03)	**<0.001**	−0.62 (0.05)	−0.65	**<0.001**	−1.51 (0.05)	**<0.001**	−0.96 (0.08)	−0.53	**<0.001**
Retirement migration	0.95 (0.07)	**<0.001**	0.24 (0.09)	0.15	**0.011**	1.66 (0.13)	**<0.001**	0.73 (0.10)	0.24	**<0.001**	2.00 (0.13)	**<0.001**	0.50 (0.12)	0.15	**<0.001**	3.23 (0.27)	**<0.001**	1.21 (0.16)	0.19	**<0.001**
Population density	−0.04 (0.00)	**<0.001**	−0.01 (0.01)	−0.10	0.150	−0.03 (0.00)	**<0.001**	−0.02 (0.01)	−0.18	**<0.001**	−0.05 (0.01)	**<0.001**	0.02 (0.01)	0.09	0.066	−0.04 (0.01)	**<0.001**	−0.01 (0.01)	−0.07	0.078
Non-white population (%)	−0.08 (0.01)	**<0.001**	0.00 (0.01)	0.00	0.940	−0.05 (0.01)	**<0.001**	0.02 (0.01)	0.09	0.055	−0.08 (0.02)	**<0.001**	0.07 (0.02)	0.15	**<0.001**	−0.05 (0.02)	**0.009**	0.09 (0.01)	0.24	**<0.001**
Constant			76.7					79.5					67.7					69.9		
r^2^			0.51					0.67					0.73					0.82		

β denotes unstandardised coefficient, Beta   denotes standardised coefficient, univariate and multivariate denote simple regression models, and univariable and multivariable denote meta-regression models.

DFLE, disability-free life expectancy; LE, life expectancy.

When all factors were included, social class, unemployment and retirement migration remained significantly associated with LE and DFLE at birth for men and women in 1991 and 2001 (the exception was retirement migration for men in 1991, which was no longer significant in the model). The multivariable model explained more of the variation in DFLE at birth (1991: women 70%, men 73%; 2001: women 81%, men 82%), than in LE at birth (1991: women 38%, men 51%; 2001: women 58%, men 67%) in both years, more of the variation in 2001 than in 1991, and more of the variation in men than for women (table 2).

In contrast, for LE and DFLE at age 85, patterns of associations across gender and time were much less clear-cut (table 3). When considered individually, social class composition and unemployment rate in an area were not significantly related to LE in 1991 or 2001, for women or men (with the exception of social class composition for men in 2001) but both were significantly and negatively related to DFLE at age 85 for both genders and time point. Retirement potential of an area was significantly and negatively related to LE at age 85 in 1991 but positively related in 2001 and ethnic composition (non-white population) was also positively associated with both LE and DFLE at age 85 in 2001. Multivariable models explained little of the variations in LE at 85 (1991: women 6%, men <1%; 2001: women 10%, men <1%) and only slightly more in DFLE at 85 (1991: women 19%, men 4%; 2001: women 47%, men 36%). Over time the relative effect (beta) decreased for unemployment but increased for all other factors. Replacing unemployment with deprivation did not substantially change the results for LE and DFLE at birth or age 85 (see online supplementary material tables S3 and S4).

**Table 3:** Explanatory simple regression and meta-regression results for LE and DFLE at age 85, 1991 and 2001, by gender

	LE age 85										DFLE age 85									
	1991					2001					1991					2001				
	Univariate		Multivariate			Univariate		Multivariate			Univariable		Multivariable			Univariable		Multivariable		
	β (SE)	p	β (SE)	Beta	p	β (SE)	p	β (SE)	Beta	p	β (SE)	p	β (SE)	Beta	p	β (SE)	p	β (SE)	Beta	p
Women																				
Social class IV and V (%)	0.01 (0.01)	0.447	0.01 (0.01)	0.10	0.211	−0.02 (0.01)	0.056	−0.01 (0.01)	−0.09	0.305	−0.02 (0.00)	**<0.001**	0.00 (0.00)	0.01	0.649	−0.04 (0.00)	**<0.001**	−0.02 (0.01)	−0.30	**<0.001**
Unemployment rate (%)	0.01 (0.01)	0.505	−0.07 (0.02)	−0.35	**<0.001**	0.01 (0.02)	0.576	0.02 (0.03)	0.08	0.456	−0.01 (0.00)	**0.001**	−0.04 (0.01)	−0.55	**<0.001**	−0.04 (0.01)	**<0.001**	0.00 (0.01)	−0.08	0.768
Retirement migration	−0.12 (0.03)	**<0.001**	−0.18 (0.05)	−0.27	**<0.001**	0.17 (0.06)	**0.003**	0.27 (0.06)	0.25	**<0.001**	0.01 (0.01)	0.426	−0.02 (0.02)	−0.10	0.331	0.23 (0.03)	**<0.001**	0.24 (0.02)	0.44	**<0.001**
Population density	0.01 (0.00)	**0.004**	0.01 (0.00)	0.25	**0.011**	0.01 (0.00)	**<0.001**	0.01 (0.00)	0.20	**0.023**	0.00 (0.00)	**<0.001**	0.01 (0.00)	0.43	**<0.001**	0.00 (0.00)	**<0.001**	0.00 (0.00)	0.27	**0.001**
Non-white population (%)	0.01 (0.01)	0.035	0.00 (0.01)	−0.02	0.81 0	0.01 (0.00)	**0.001**	0.01 (0.01)	0.08	0.325	0.01 (0.00)	**<0.001**	0.00 (0.00)	0.10	0.231	0.01 (0.00)	**<0.001**	0.01 (0.00)	0.24	**<0.001**
Constant			6.85					6.09					1.71					1.39		
r^2^			0.06					0.09					0.19					0.47		
Men																				
Social class IV and V (%)	−0.01 (0.01)	0.306	−0.01 (0.01)	−0.07	0.362	−0.04 (0.01)	**<0.001**	−0.04 (0.01)	−0.26	**0.004**	−0.02 (0.00)	**<0.001**	−0.01 (0.01)	−0.09	0.089	−0.04 (0.00)	**<0.001**	−0.02 (0.01)	−0.32	**<0.001**
Unemployment rate (%)	0.00 (0.01)	0.926	−0.02 (0.02)	−0.09	0.372	−0.02 (0.02)	0.260	0.04 (0.03)	0.11	0.295	−0.02 (0.01)	**0.002**	−0.02 (0.01)	−0.22	**0.004**	−0.05 (0.01)	**<0.001**	−0.02 (0.01)	−0.12	**0.077**
Retirement migration	−0.05 (0.04)	0.212	−0.10 (0.05)	−0.13	0.070	0.16 (0.06)	**0.007**	0.19 (0.06)	0.17	**0.003**	0.02 (0.02)	0.153	−0.01 (0.02)	−0.06	0.579	0.19 (0.03)	**<0.001**	0.16 (0.03)	0.30	**<0.001**
Population density	0.00 (0.00)	0.188	0.00 (0.00)	0.09	0.344	0.01 (0.00)	**0.001**	0.00 (0.00)	0.11	0.232	0.00 (0.00)	0.204	0.00 (0.00)	0.09	0.065	0.00 (0.00)	**0.001**	0.00 (0.00)	0.18	0.032
Non-white population (%)	0.01 (0.01)	0.366	0.00 (0.01)	−0.02	0.760	0.01 (0.00)	**0.001**	0.01 (0.01)	0.12	0.125	0.00 (0.00)	0.08	0.00 (0.00)	0.09	0.136	0.01 (0.00)	**<0.001**	0.01 (0.00)	0.23	**<0.001**
Constant			5.48					5.56					1.89					1.79		
r^2^			0.01					0.1					0.04					0.36		

β denotes unstandardised coefficient, Beta denotes standardised coefficient, univariate and multivariate denote simple regression models, and univariable and multivariable denote meta-regression models.

DFLE, disability-free life expectancy; LE, life expectancy.

## Discussion

Our comparison of changes over time (between 1991 and 2001) in LE and DFLE values at birth and at age 85 contributes to our understanding of the widening differentials in health within GB identified by the Marmot Review.^4^ Although average values and gaps between local areas in LE and DFLE at age 85 changed similarly to those at birth, geographic variation, in terms of the northwest southeast divide, urban–rural or deprivation gradient were less apparent at age 85 than at birth. Moreover, the factors explaining inequalities between local areas in LE and DFLE at birth mostly did not explain inequalities at age 85.

We found that between 1991 and 2001 and across GB local areas, LE and DFLE at birth and age 85 increased, but LE to a larger extent than DFLE. While the gap across LAs generally increased for both indicators (though the gap in LE at age 85 decreased), the gap in DFLE increased more than that in LE. Patterns of LE and DFLE at birth—northwest southeast divide, urban to rural as well as for the deprivation divide—persisted over time between 1991 and 2001. We observe clear declines in these measures as deprivation increases across LAs and the decline is steeper with DFLE where morbidity is added to mortality. For the very old, the northwest southeast divide is much less apparent and the relationship with deprivation disappears for LE though is still present for DFLE as is the greater gains in less-deprived areas. That gains in LE and DFLE at birth are greater in less-deprived quintiles might result from public health interventions being more successful for the affluent and healthier population or less effective for people in lower socioeconomic status,^25^ ^26^ but, as our analysis is at an area level, migration (eg, healthier people moving to already ‘healthier areas’) may also have an effect.^27^

All these findings show that LE as a population health indicator produces a more positive picture of population health (at age 85 as well as at birth) than DFLE, confirming other studies.^3^ ^6^ This is especially important, as within England the Department of Health formula to allocate National Health Service (NHS) funds presently uses standardised mortality ratio (SMR) <75 (closely related to LE) rather than DFLE^28^ and this will not necessarily allocate sufficient funds to areas most in need.

Health inequalities are manifested by various socioeconomic, demographic and environmental variables. We found that social class composition, unemployment rate, retirement potential, population density and ethnic composition explain more variation in DFLE across areas than variation in LE, slightly more of the variation in men than in women, and more of the variation in 2001 than in 1991. However, the relationship of LE and DFLE at birth with unemployment rates changed over time. Unemployment rates were higher and discriminated sharply between areas in 1991, while in 2001 they were lower and less discriminatory. Overall, there is a stronger relationship between social class and deprivation and LE and DFLE in 2001 compared with 1991. Indeed, social class composition is more important in 2001 than in 1991 when unemployment rate was the most important factor.

Bone *et al* 1995^18^ hypothesised a negative relationship between the percentages of non-white population in an area to DFLE. In contrast, both their 1995 study and ours observe a marginal positive relationship if all other area variables are considered simultaneously, although because deprivation is controlled for we may be picking up a small healthy migrant effect. In future we might be able to test this hypothesis by using census information on the number of first-generation migrants living in an area, which is not available for 1991.

Limitations of our study include the need to harmonise the limiting longstanding illness question in 1991 and 2001 and other potential explanatory factors we have not considered. On a national level, harmonised data reproduced Health Survey for England DFLE 1991, which used the 2001 census question. Harmonised data were, as anticipated, lower but differences decreased with age, which is plausible as older people may be less likely to under-report illness in 1991. Education has often been identified as an important factor influencing positively DFLE.^29^ However, from the 1991 and 2001 censuses, comparable information is available only on the number of persons with a degree qualification in an area, a rather blunt measure of educational level. We included this variable but it did not improve the model fit and therefore we excluded it from reporting. The strengths of our study are the more rigorous analytic methods of drivers of inequalities over the previous study,^18^ including alignment of 1991 and 2001 geographies to account for boundary changes, use of all LAs in England and Wales and meta-regression methods for DFLE that incorporates uncertainty around DFLE estimates.

Our study suggests inequalities in LE and DFLE at age 85, as those at birth, persist and have increased for DFLE as a result of larger gains in more affluent areas. We also suggest that factors explaining variation in LE at birth between areas may not be the same as those that explain underlying differences in DFLE at birth and in both cases these may not explain inequalities at very old ages. Given the greater disability and illness burden in very late life as well as the continued growth in this age group, monitoring inequalities in health expectancies across local areas at very old ages, as well as at birth, and understanding their drivers will be key to achieve the desired target of health for all.

**What is already known on this subject?**

- Area variation in health and mortality across Great Britain (GB) local areas is well established as are relationships between deprivation and less good health, and an urban rural gradient.
- To date, no comparison in disability-free life expectancy (DFLE) over time on a local area level has been done.
- Published studies have usually focused on at birth and age 65 populations, and not on the very old, a very fast growing segment of the GB population.

**What this study adds?**

- Drivers for DFLE variation at birth do not explain DFLE variation in the very old.
- With regards to health improvement, not only are less affluent areas being left behind but worryingly DFLE may even be decreasing at older ages in more-deprived areas.
- Life expectancy (LE) does not reflect the full extent of health inequality in GB, by using LE as a health indicator, conditions look better than they are and areas in need might not get the support they need.

## Supplementary Material

Web supplement
