# Supplementary material for: Drivers of inequality in disability-free expectancy at birth and age 85 across space and time in Great Britain
Source: J Epidemiol Community Health. 2014 Jun 6;68(9):826–33. doi: 10.1136/jech-2014-204083 (PMC4145463; doi:10.1136/jech-2014-204083)
Supplement: Web supplement [file jech-2014-204083-s1.pdf]

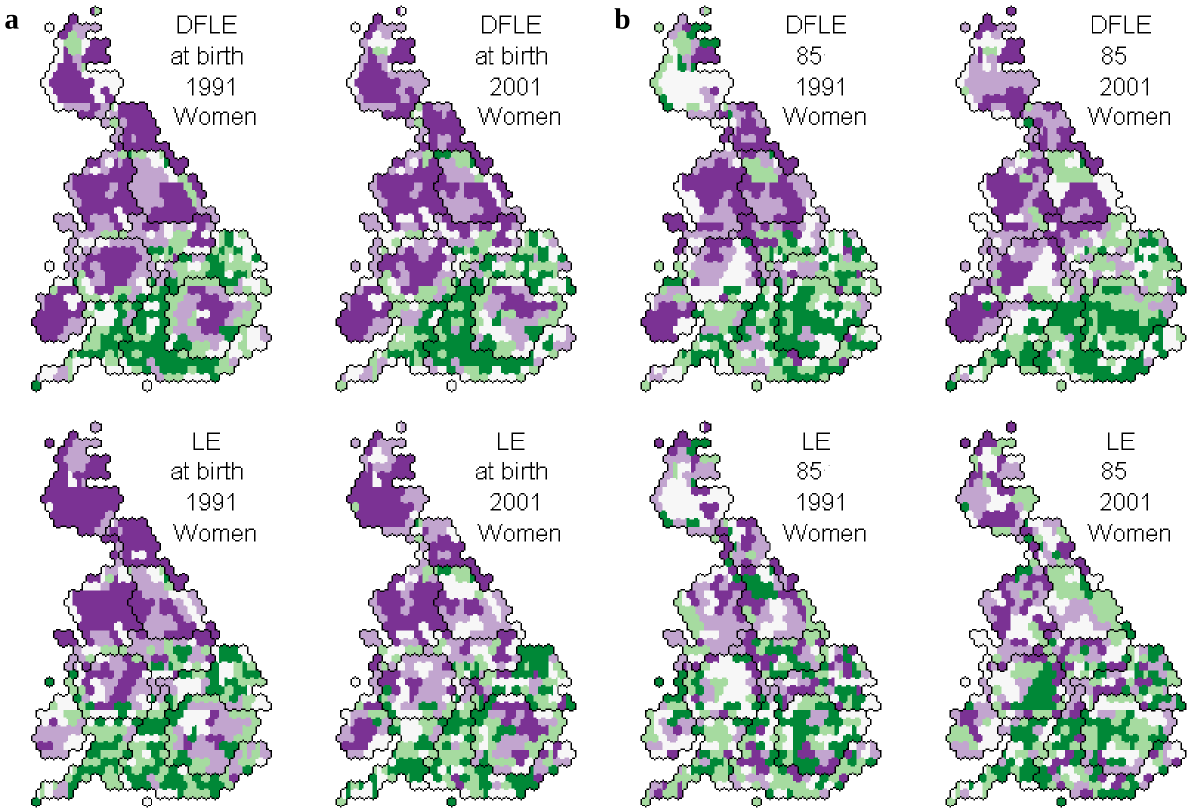

SOM Figure 1 DFLE and LE area quintiles, 1991 and 2001, women at birth (a) and age 85 (b). GB local areas presented in a population cartogram to highlight population numbers affected. Each hexagon denotes half a parliamentary constituency.

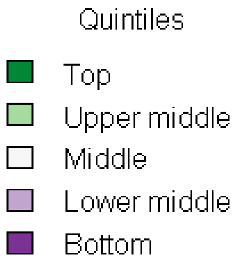

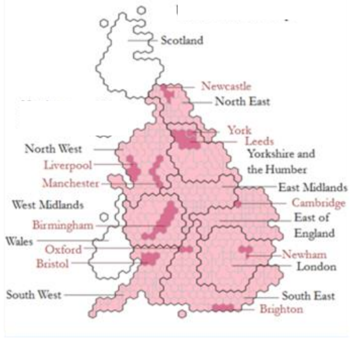

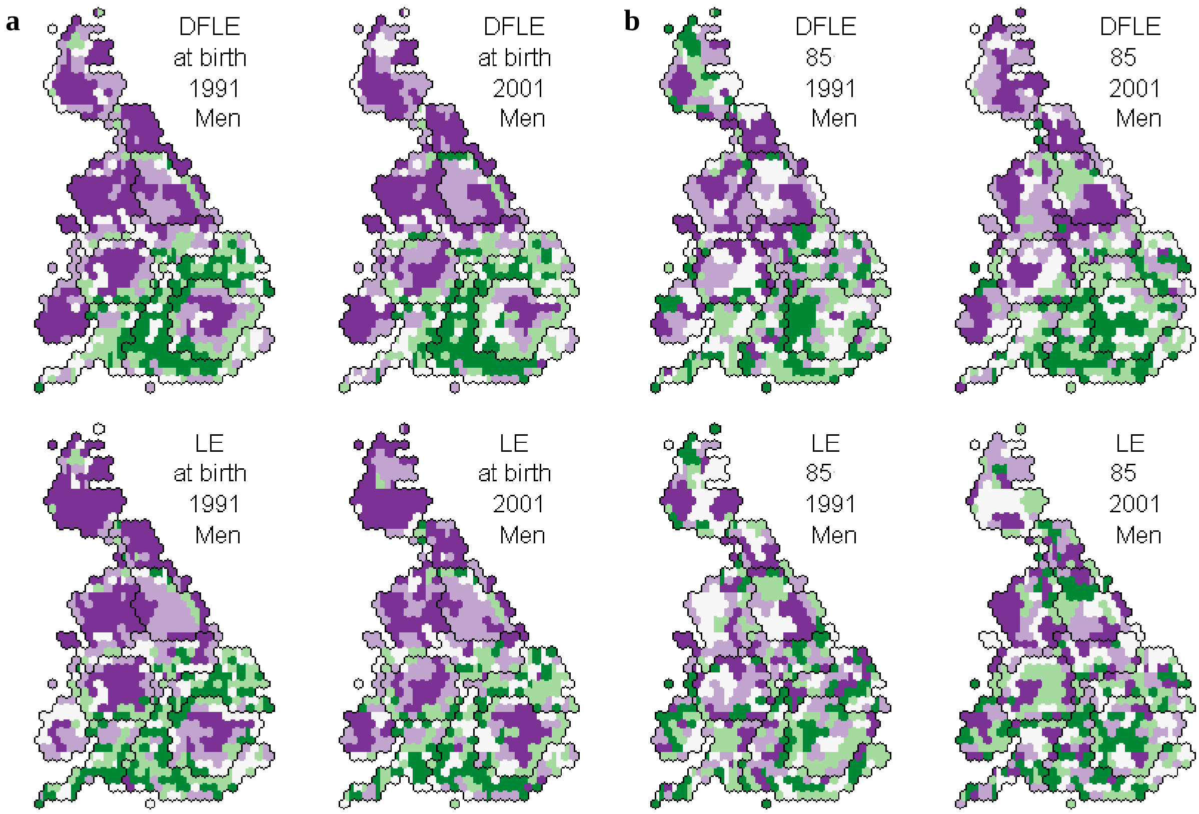

SOM Figure 2 DFLE and LE quintiles, 1991 and 2001, men at birth (a) and 85 (b). GB local areas.

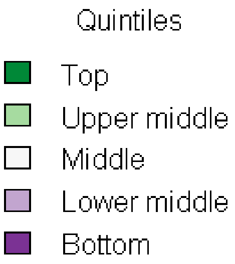

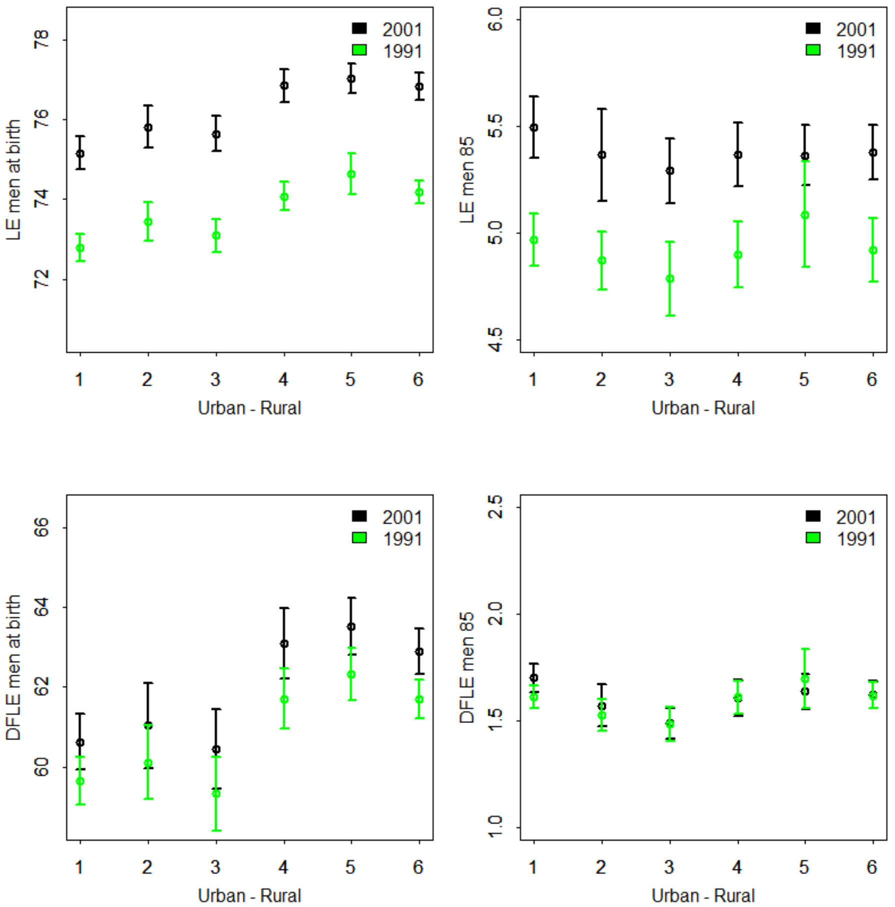

SOM Figure 3 Mean life expectancy (LE) and mean disability free life expectancy (DFLE), men, at birth and age 85, 1991 and 2001, by urban – rural classification. Note: 1= most urban, 6=most rural

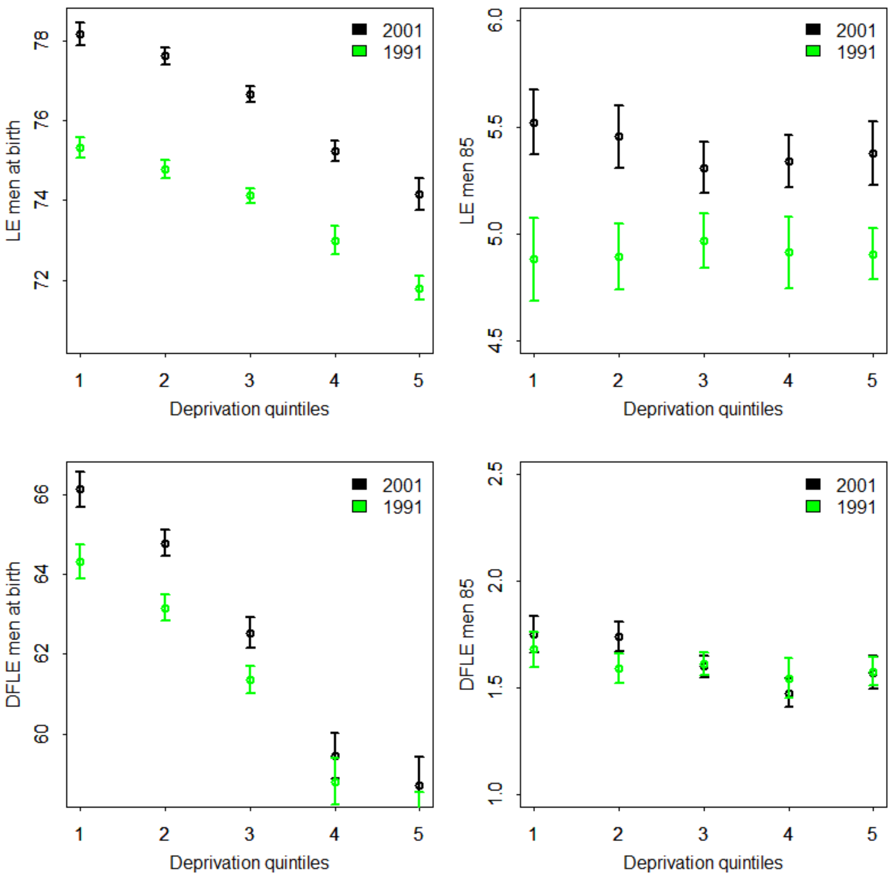

SOM Figure 4 Mean life expectancy (LE) and disability free life expectancy (DFLE), men, at birth and 85, 1991 and 2001, by deprivation quintiles applying the Townsend score 2001. Note: 1=most affluent, 2=most deprived.

SOM Table 1a: Population density classes adapted from DEFRA

Density Classes	DEFRA Classification name	Population density (Person per hectare)
1	Major Urban	≥22.57 to 133.72
2	Large Urban	≥11.81 to < 22.57
3	Other Urban	≥4.99 to < 11.81
4	Significant Rural	≥2.71 to < 4.99
5	Rural-50	≥1.54 to < 2.71
6	Rural-80	<1.54

SOM Table 1b: Area characteristics

Parameter	Details
Unemployment rate	as the percentage of all economically active persons who are unemployed
Social class composition	as the percentage of people in households in social class IV or V of all people living in economically active households (defined by household head)
Ethnic composition	as the percentage of all persons defining themselves other than White
Retirement migration potential	as the percentage of people of pensionable age who moved into an area throughout the last year
Deprivation	Townsend deprivation index score
Population density	Person per hectare

Notes: All data adapted to be equivalent between 1991 and 2001

SOM Table 2 Regression results of life expectancy (LE) and disability free life expectancy (DFLE) at birth and age 85, by gender and their relationship over time with the urban-rural gradient or deprivation quintiles

			LE				DFLE			
			**Women**		**Men**		**Women**		**Men**	
			β (SE)	p	β (SE)	p	β (SE)	p	β (SE)	p
**With interaction term**	**at birth**	**Urban-rural**	0.13 (0.04)	**<0.000**	0.32 (0.05)	**<0.000**	0.49 (0.08)	**<0.000**	0.52 (0.09)	**<0.000**
		**Time**	1.35 (0.21)	**<0.000**	2.35 (0.26)	**<0.000**	0.40 (0.44)	0.362	0.94 (0.49)	0.058
		**Urban-rural*time**	0.10 (0.05)	0.057	0.05 (0.06)	0.463	0.07 (0.11)	0.500	0.05 (0.12)	0.663
	**at 85**	**Urban-rural**	-0.06 (0.02)	**0.001**	0.01 (0.02)	0.620	-0.01 (0.01)	0.471	0.02 (0.01)	0.090
		**Time**	0.15 (0.1)	0.127	0.55 (0.1)	**0.000**	-0.01 (0.04)	0.841	0.08 (0.05)	0.100
		**Urban-rural*time**	0.02 (0.02)	0.468	-0.02 (0.03)	0.334	0.00 (0.01)	0.984	-0.02 (0.01)	0.127
**No interaction term**	**at birth**	**Urban-rural**	0.18 (0.03)	**<0.000**	0.34 (0.03)	**<0.000**	0.53 (0.05)	**<0.000**	0.54 (0.06)	**<0.000**
		**Time**	1.72 (0.1)	**<0.000**	2.52 (0.12)	**<0.000**	0.66 (0.2)	**0.001**	1.13 (0.22)	**<0.000**
	**at 85**	**Urban-rural**	-0.05 (0.01)	**<0.000**	0.00 (0.01)	0.790	-0.01 (0.01)	0.318	0.01 (0.01)	0.384
		**Time**	0.21 (0.04)	**<0.000**	0.46 (0.05)	**<0.000**	-0.01 (0.02)	0.685	0.01 (0.02)	0.531
**With interaction term**	**at birth**	**Deprivation**	-0.55 (0.04)	**<0.000**	-0.82 (0.04)	**<0.000**	-1.34 (0.07)	**<0.000**	-1.60 (0.08)	**<0.000**
		**Time**	2.13 (0.18)	**<0.000**	2.97 (0.2)	**<0.000**	1.60 (0.33)	**<0.000**	1.96 (0.36)	**<0.000**
		**Deprivation*time**	-0.14 (0.05)	**0.013**	-0.15 (0.06)	**0.016**	-0.31 (0.1)	**0.002**	-0.28 (0.11)	**0.012**
	**at 85**	**Deprivation**	0.03 (0.02)	0.198	0.01 (0.02)	0.516	-0.02 (0.01)	0.069	-0.02 (0.01)	0.127
		**Time**	0.28 (0.1)	**0.008**	0.6 (0.11)	**<0.000**	0.09 (0.05)	0.057	0.15 (0.05)	**0.005**
		**Deprivation*time**	-0.02 (0.03)	0.489	-0.04 (0.03)	0.166	-0.03 (0.01)	**0.022**	-0.04 (0.02)	**0.006**
**No interaction term**	**at birth**	**Deprivation**	-0.61 (0.03)	**<0.000**	-0.89 (0.03)	**<0.000**	-1.50 (0.05)	**<0.000**	-1.74 (0.06)	**<0.000**
		**Time**	1.72 (0.08)	**<0.000**	2.52 (0.09)	**<0.000**	0.66 (0.14)	**<0.000**	1.13 (0.16)	**<0.000**
	**at 85**	**Deprivation**	0.02 (0.02)	0.259	-0.01 (0.02)	0.640	-0.03 (0.01)	**<0.000**	-0.04 (0.01)	**<0.000**
		**Time**	0.21 (0.04)	**<0.000**	0.46 (0.05)	**<0.000**	-0.01 (0.02)	0.681	0.01 (0.02)	0.525

SOM Table 3 Explanatory regression and meta-regression results for LE and DFLE at birth, 1991 and 2001, by gender

		**LE at birth**										**DFLE at birth**									
		**1991**					**2001**					**1991**					**2001**				
		Univariate		Multivariate			Univariate		Multivariate			Univariable		Multivariable			Univariable		Multivariable		
		β<br>(SE)	p	β<br>(SE)	Beta	p	β<br>(SE)	p	β<br>(SE)	Beta	p	β<br>(SE)	p	β<br>(SE)	Beta	p	β<br>(SE)	p	β<br>(SE)	Beta	p
**Women**	**Social Class IV and V (%)**	-0.17<br>(0.01)	**<0.001**	-0.07<br>(0.02)	-0.22	**<0.001**	-0.24<br>(0.01)	**<0.001**	-0.14<br>(0.02)	-0.41	**<0.001**	-0.46<br>(0.02)	**<0.001**	-0.17<br>(0.03)	-0.25	**<0.001**	-0.66<br>(0.02)	**<0.001**	-0.39<br>(0.03)	-0.48	**<0.001**
	**Deprivation**	-0.19<br>(0.02)	**<0.001**	-0.22<br>(0.04)	-0.60	**<0.001**	-0.23<br>(0.02)	**<0.001**	-0.11<br>(0.04)	-0.27	**0.002**	-0.53<br>(0.03)	**<0.001**	-0.56<br>(0.07)	-0.73	**<0.001**	-0.63<br>(0.03)	**<0.001**	-0.43<br>(0.06)	-0.46	**<0.001**
	**Retirement migration**	0.56<br>(0.06)	**<0.001**	0.01<br>(0.08)	0.01	**0.860**	1.36<br>(0.10)	**<0.001**	0.76<br>(0.10)	0.31	**<0.001**	1.71<br>(0.11)	**<0.001**	0.34<br>(0.12)	0.12	**0.02**	3.18<br>(0.24)	**<0.001**	1.36<br>(0.15)	0.24	**<0.001**
	**Population density**	-0.01<br>(0.00)	**<0.001**	0.01<br>(0.01)	0.19	**0.020**	-0.02<br>(0.00)	**<0.001**	0.00<br>(0.01)	0.06	0.396	-0.04<br>(0.01)	**<0.001**	0.03<br>(0.01)	0.18	**0.012**	-0.04<br>(0.01)	**<0.001**	0.01<br>(0.01)	0.04	0.903
	**Non-white population (%)**	-0.03<br>(0.01)	**0.006**	0.02<br>(0.01)	0.13	0.060	-0.03<br>(0.01)	**<0.001**	0.01<br>(0.01)	0.07	0.189	-0.08<br>(0.02)	**0.001**	0.08<br>(0.02)	0.21	**<0.001**	-0.07<br>(0.02)	**<0.001**	0.08<br>(0.01)	0.21	**<0.001**
	**Constant**			80.11					82.12					65.28					68.49		
	**r^2**			0.37					0.58					0.66					0.8		
**Men**	**Social Class IV and V (%)**	-0.22<br>(0.02)	**<0.001**	-0.09<br>(0.02)	-0.22	**<0.001**	-0.31<br>(0.02)	**<0.001**	-0.16<br>(0.02)	-0.37	**<0.001**	-0.54<br>(0.03)	**<0.001**	-0.18<br>(0.03)	-0.24	**<0.001**	-0.73<br>(0.03)	**<0.001**	-0.37<br>(0.03)	-0.41	**<0.001**
	**Deprivation**	-0.32<br>(0.02)	**<0.001**	-0.24<br>(0.05)	-0.52	**<0.001**	-0.35<br>(0.02)	<0.001	-0.22<br>(0.04)	-0.44	**<0.001**	-0.63<br>(0.03)	**<0.001**	-0.71<br>(0.07)	-0.79	**<0.001**	-0.71<br>(0.04)	**<0.001**	-0.65<br>(0.06)	-0.63	**<0.001**
	**Retirement migration**	0.95<br>(0.07)	**<0.001**	0.18<br>(0.09)	0.11	0.050	1.66<br>(0.13)	**<0.001**	0.67<br>(0.10)	0.23	**<0.001**	2.00<br>(0.13)	**<0.001**	0.37<br>(0.13)	0.11	**0.014**	3.23<br>(0.27)	**<0.001**	1.09<br>(0.16)	0.17	**<0.001**
	**Population density**	-0.04<br>(0.00)	**<0.001**	0.00<br>(0.01)	-0.02	0.790	-0.03<br>(0.00)	**<0.001**	-0.01<br>(0.01)	-0.06	0.317	-0.05<br>(0.01)	**<0.001**	0.03<br>(0.01)	0.16	**0.014**	-0.04<br>(0.01)	**<0.001**	0.01<br>(0.01)	0.05	0.702
	**Non-white population (%)**	-0.08<br>(0.01)	**<0.001**	0.02<br>(0.02)	0.10	0.100	-0.05<br>(0.01)	**<0.001**	0.03<br>(0.01)	0.16	**0.001**	-0.08<br>(0.02)	**0.003**	0.14<br>(0.02)	0.29	**<0.001**	-0.05<br>(0.02)	**0.032**	0.13<br>(0.01)	0.34	**<0.001**
	**Constant**			74.86					78					62.36					65.71		
	**r^2**			0.51					0.68					0.7					0.81		

Notes: β = unstandardized coefficient, Beta=Standardised coefficient, Univariate and Multivariate denote simple regression models, Univariable and Multivariable denote meta-regression models

SOM Table 4 Explanatory simple regression and meta-regression results for LE and DFLE at age 85, 1991 and 2001, by gender

		LE at birth										DFLE at birth									
		1991					2001					1991					2001				
		Univariate		Multivariate			Univariate		Multivariate			Univariable		Multivariable			Univariable		Multivariable		
		β (SE)	p	β (SE)	Beta	p	β (SE)	p	β (SE)	Beta	p	β (SE)	p	β (SE)	Beta	p	β (SE)	p	β (SE)	Beta	p
Women	**Social Class IV and V (%)**	0.01 (0.01)	0.447	0.01 (0.01)	0.06	0.460	-0.02 (0.01)	**0.056**	-0.03 (0.01)	-0.18	**0.028**	-0.02 (0.00)	**<0.001**	-0.01 (0.00)	-0.07	**0.635**	-0.04 (0.00)	**<0.001**	-0.04 (0.01)	-0.47	**<0.001**
	**Deprivation**	0.02 (0.01)	**0.046**	-0.06 (0.03)	-0.33	**0.020**	0.02 (0.01)	**0.018**	0.05 (0.02)	0.29	**0.025**	0.00 (0.00)	**0.926**	-0.03 (0.01)	-0.45	**0.004**	-0.01 (0.01)	**0.475**	0.02 (0.01)	0.25	**0.046**
	**Retirement migration**	-0.12 (0.03)	**<0.001**	-0.18 (0.05)	-0.27	**0.000**	0.17 (0.06)	**0.003**	0.29 (0.06)	0.27	**<0.001**	0.01 (0.01)	**0.841**	-0.02 (0.02)	-0.08	**0.686**	0.23 (0.03)	**<0.001**	0.25 (0.02)	0.47	**<0.001**
	**Population density**	0.01 (0.00)	**0.004**	0.01 (0.00)	0.24	**0.020**	0.01 (0.00)	**<0.001**	0.00 (0.00)	0.07	0.484	0.00 (0.00)	**<0.001**	0.01 (0.00)	0.38	**<0.001**	0.00 (0.00)	**<0.001**	0.00 (0.00)	0.10	0.657
	**Non-white population (%)**	0.01 (0.01)	**0.035**	0.00 (0.01)	0.04	0.660	0.01 (0.00)	**0.001**	0.00 (0.01)	0.03	0.679	0.01 (0.00)	**0.001**	0.01 (0.00)	0.17	**0.117**	0.01 (0.00)	**<0.001**	0.01 (0.00)	0.2	**0.003**
	**Constant**			6.37					5.91					1.46					1.59		
	**r^2**			0.05					0.11					0.13					0.47		
Men	**Social Class IV and V (%)**	-0.01 (0.01)	0.306	-0.03 (0.01)	-0.16	0.070	-0.04 (0.01)	**<0.001**	-0.05 (0.01)	-0.32	**<0.001**	-0.02 (0.00)	**<0.001**	-0.01 (0.01)	-0.15	**0.099**	-0.04 (0.00)	**<0.001**	-0.04 (0.01)	-0.45	**<0.001**
	**Deprivation**	0.01 (0.01)	0.391	0.02 (0.03)	0.10	0.510	0.01 (0.01)	0.449	0.05 (0.02)	0.27	**0.042**	-0.01 (0.01)	**0.242**	-0.02 (0.01)	-0.13	**0.385**	-0.01 (0.01)	**0.038**	0.00 (0.01)	0.11	**0.773**
	**Retirement migration**	-0.05 (0.04)	0.212	-0.07 (0.05)	-0.10	0.200	0.16 (0.06)	**0.007**	0.21 (0.06)	0.18	**0.001**	0.02 (0.02)	**0.342**	-0.01 (0.02)	-0.05	**0.894**	0.19 (0.03)	**<0.001**	0.17 (0.03)	0.32	**<0.001**
	**Population density**	0.00 (0.00)	0.188	0.00 (0.00)	0.00	0.970	0.01 (0.00)	**0.001**	0.00 (0.00)	0.01	0.956	0.00 (0.00)	**0.442**	0.00 (0.00)	0.05	**0.642**	0.00 (0.00)	**0.002**	0.00 (0.00)	0.07	0.912
	**Non-white population (%)**	0.01 (0.01)	0.366	-0.01 (0.01)	-0.05	0.570	0.01 (0.00)	**0.001**	0.01 (0.01)	0.08	**0.322**	0.00 (0.00)	**0.187**	0.01 (0.00)	0.11	**0.263**	0.01 (0.00)	**<0.001**	0.01 (0.00)	0.22	**0.002**
	**Constant**			5.56					0					1.74					1.85		
	**r^2**			0					0					0.03					0.35		

Notes: β = unstandardized coefficient, Beta=Standardised coefficient, Univariate and Multivariate denote simple regression models, Univariable and Multivariable denote meta-regression models

- **1991**
- ❑ Do you have any long-term illness, health problem or handicap which limits your daily activities or the work you can do? Include problems which are due to old age.
- ❑ Yes, I have a health problem which limits activities
- ❑ I have no such health problem
- **2001**
- ❑ Do you have any long-term illness, health problem or disability which limits your daily activities or the work you can do? Include problems which are due to old age.
- ❑ Yes
- ❑ No

SOM Box 1 1991 and 2001 UK Census questions on long-term illness
